# Transition metal driven altermagnetism and spin–orbit coupling effect in tetragonal Ce-based pnictides: a first-principles investigation

**DOI:** 10.1039/d6ra02985j

**Published:** 2026-05-26

**Authors:** Narayanan Namboodiri Puthusseri, Sambit Jena, Tanay Nag, Banasree Sadhukhan, Pankaj Bhalla

**Affiliations:** a Department of Physics, School of Engineering and Sciences, SRM University AP Amaravati 522240 India pankaj.b@srmap.edu.in; b Department of Physics, École Centrale School of Engineering, Mahindra University Hyderabad Telangana 500043 India; c Department of Physics, BITS Pilani-Hyderabad Campus Telangana 500078 India

## Abstract

We present a first-principles density functional theory (DFT) investigation of the structural, electronic and magnetic properties of the tetragonal CeX_2_Y_2_ (X = Co, Ni, and Fe; Y = P, As, and Sb) compounds, crystallizing in the ThCr_2_Si_2_-type structure (space group *I*4/*mmm*). Electronic properties of the relaxed structure are analyzed, highlighting the influence of Ce-4f and Co-3d states on the magnetic behaviour. The studies reveal that the Fe and Ni based compounds exhibit weak magnetism, and the Co-based compounds follow interlayer antiferromagnetic behaviour. The calculated density of states reveals a significant contribution from Ce-4f and Co-3d orbitals near the Fermi level (*E*_F_), indicating a complex interplay between localized and itinerant electronic states. The results further suggest the potential realization of altermagnetism arising from the magnetic structure of the CeCo_2_P_2_, showing d-wave like band splitting in the band structure and four lobe structure in the Fermi surface picture. Inclusion of spin–orbit coupling (SOC) leads to noticeable band splitting and modification of the near-Fermi-level band dispersion, which is relevant for understanding relativistic effects in Ce-based intermetallic compounds. These results provide a reliable computational baseline for further investigations of magnetism and possible topological features in CeX_2_Y_2_ and related materials. The present study serves as a starting point toward exploring correlation-driven and spin–orbit–induced phenomena in Ce-based pnictides.

## Abstract 2

The study reveals a momentum-dependent spin splitting in CeCo_2_P_2_, indicating a potential d-wave altermagnetic behaviour. These findings highlight its promise for applications in next-generation spintronic devices.

## Graphical abstract

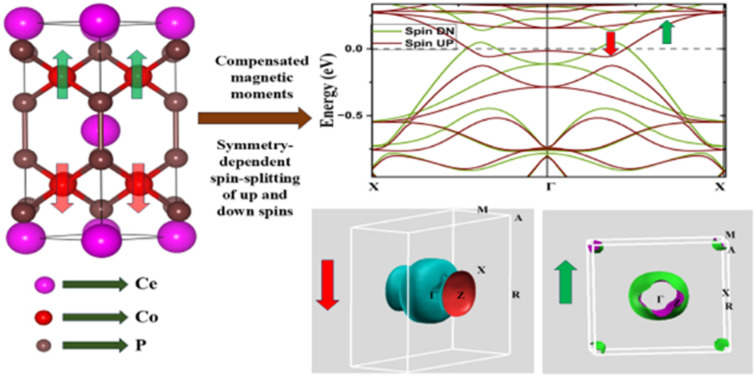

## 1 Introduction

Rare-earth-based compounds constitute a fertile platform for exploring strongly correlated electron physics, where the interplay between localized 4f states and itinerant conduction electrons gives rise to a wide spectrum of emergent phenomena.^1–5^ Among these materials, cerium-based systems occupy a central position due to the unique electronic configuration of Cerium (Ce), whose partially filled 4f states lie energetically close to the Fermi level (*E*_F_). This enables an exquisite competition between localization and itinerancy, giving rise to mixed-valence behavior, Kondo screening, heavy-fermion characteristics, magnetically ordered phases, and in certain cases, unconventional superconductivity.^6–12^ Cerium bismuthide (CeBi) represents an important subclass of rare-earth monopnictides in which magnetism, electronic structure, and lattice symmetry are strongly interrelated.^13,14^ Their physical behaviour is governed by competition among exchange interactions, crystal-field splitting, spin–orbit coupling (SOC), and 4f–p hybridization.^15^ While the cubic rock-salt phase of Ce monopnictides has been extensively examined both experimentally and theoretically,^16–18^ considerably less attention has been devoted to tetragonal phases. Importantly, the reduced symmetry from highly symmetric cubic phase to tetragonal symmetry can fundamentally alter the electronic dispersion, lift degeneracies, enhance magnetic anisotropy, and modify exchange pathways.^19^

The ThCr_2_Si_2_-type tetragonal structure (space group – *I*4/*mmm*) has long served as a versatile structural framework for hosting correlated electron systems.^20^ The layered architecture, comprising a transition metal–pnictogen slab and structure separated by rare-earth planes, naturally promotes anisotropic electronic behavior and competing magnetic interactions. The Cobalt (Co) atoms at different layers are found to interact antiferromagnetically, forming an A-type antiferromagnetic (AFM) structure. Recent studies have found CeCo_2_P_2_, a unique topological heavy-fermion material where new quantum phases are produced by the interaction of quantum geometry, band topology, the Kondo effect, and magnetism.^21,22^ Within this family of cerium-based compounds, two distinct types of electrons are present, the migrant c electrons and the strongly correlated Ce-f electrons. The itinerant electrons arise from the p orbitals of Phosphorus (P) and the d orbitals of Co and Ce. The Ce 4f electron can be found where the itinerant and localised regimes meet, making these materials highly sensitive to lattice symmetry, chemical bonding, and hybridization strength.^21^

Recently, Resham *et al.* have identified altermagnetic behavior in Co intercalated CoNb_4_Se_8_ and the DFT studies confirmed A-type AFM-ordering in this compound.^23^ Altermagnetism is a recently discovered magnetic phase that is fundamentally different from both ferromagnets and antiferromagnets but incorporates features of both.^24,25^ Like conventional antiferromagnets, altermagnets show complete compensation of magnetic moments on different sublattices, leading to zero net magnetization however, they exhibit a distinctive momentum dependent spin splitting in their electronic band structure, arising from crystal symmetry mainly, rotational symmetry rather than from net magnetic order. As a result, new transport and spintronic functions are made possible by spin-polarized electronic states even in the absence of macroscopic magnetisation. Their strong spin-dependent electronic responses and reduced sensitivity to stray magnetic fields, establishing altermagnets as a distinct class of magnetic materials for spintronic applications.^26–30^ In the present work, the antiferromagnetic coupling of Co atoms in CeCo_2_P_2_ attracts interest for investigating the novel altermagnetic phenomenon. Given the recent studies on Co- and Ce-based compounds, understanding the structural stability and magnetic ground states of tetragonal Ce-based pnictides is very important. This can be attributed to the following reasons. First, symmetry lowering can lift band degeneracies and alter Fermi surface topology, potentially enabling anisotropic transport and magnetocrystalline effects. Second, the SOC inherent to Ce combined with p-orbital hybridization from heavier pnictogens may produce non-trivial electronic features near the Fermi level, with possible implications for correlated and topological phases.

In this work, we present a systematic first-principles study of the structural, electronic, and magnetic properties of tetragonal Ce-based CeX_2_Y_2_ (X = Co, Ni, and Fe; Y = P, As, and Sb) pnictides. Among the studied compounds CeCo_2_P_2_, CeCo_2_As_2_, CeFe_2_P_2_, and CeNi_2_P_2_ have been experimentally realised, whereas no studies have been reported for CeCo_2_Sb_2_.^31–37^ Structural optimization is performed to determine equilibrium lattice parameters and assess phase stability. The electronic band structure (BS) and density of states (DOS) are analyzed to identify the role of Ce 4f states and their hybridization with pnictogen p orbitals. Different magnetic configurations are examined to identify the magnetic ground state and quantify local magnetic moments. From the obtained results, it was seen that the Co atoms couple antiferromagnetically, exhibiting A-type antiferromagnetic behaviour, whereas the magnetic moments at the Fe and Ni sites are zero. Further, the CeCo_2_Y_2_ systems were analysed for altermagnetic behaviour due to the A-type AFM ordering and CeCo_2_P_2_ is found to exhibit altermagnetic splitting along the high symmetry k-path and the Fermi surface analysis reveals d-wave altermagnetism in CeCo_2_P_2_. Further to explore the band splitting, the effect of SOC has been considered, suggesting that the SOC-induced relativistic effects in the Ce-based pnictides lift the degeneracy.

## 2 Computational techniques

Using the plane-wave implementation of the Quantum ESPRESSO package, spin-polarized density functional theory (DFT) calculations were carried out in this work. Projector-Augmented Wave (PAW) pseudopotentials were used to describe electron–ion interactions,^38–40^ with the valence electron configurations are treated as Fe (3d^6^,4s^2^), Co (3d^7^,4s^2^), Ni (3d^8^,4 s^2^), P (3s^2^,3p^3^), As (4s^2^,4p^3^), Sb (5s^2^,5p^3^). The exchange–correlation effects were treated within the framework of the generalized gradient approximation (GGA) using the Perdew–Burke–Ernzerhof (PBE) functional.^41^ The structure is completely optimized using vc-relax, and the crystal is fully relaxed in both non-magnetic and magnetic configurations. A force convergence value of 10^−3^ eV Å^−1^ has been set between the adjacent atoms. The self-consistent field (SCF) calculations are run for a total energy convergence of 1 × 10^−7^ eV, and an energy cutoff of 820 eV has been used for the plane wave expansion. In order to consider the strong correlation effect in transition metals, as well as the rare-earth element Ce, we have included the Hubbard parameter, *U*, along with GGA-PBE for a better understanding of the materials. The *U* value used for Ce, *U*_*Ce*_ = 5 eV^42^ and for Co, *U*_*Co*_ = 2.7 eV.^43^ For sampling the Brillouin zone, a Γ-centered *k*-point mesh of 12 × 12 × 8 is chosen, and the electronic occupations are treated using the Methfessel–Paxton (MP) smearing scheme with a smearing width of 0.27 eV to improve convergence for the metallic states.^44^ Furthermore, the effect of SOC has been incorporated into the system within the DFT framework.

## 3 Results and discussions

### 3.1 Structural details

**Fig. 1:**
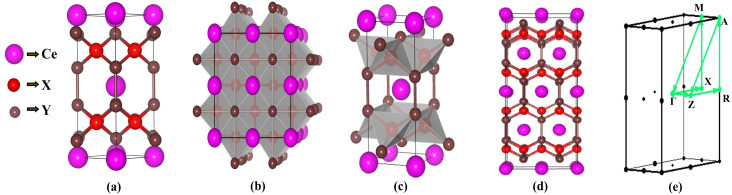
(a) Crystal structure of CeX_2_Y_2_ (X = Fe, Co, and Ni; Y = P, As and Sb) in the tetragonal ThCr_2_Si_2_-type structure, (b) edge-sharing XY_4_ tetrahedra, (c) top view of CeX_2_Y_2_ showing the square-planar arrangement of X atoms, (d) front view of CeX_2_Y_2_, and (e) high symmetry k-path used for plotting band structure.

The CeX_2_Y_2_ compound was modelled in the tetragonal ThCr_2_Si_2_-type structure with space group *I*4/mmm, consistent with experimentally reported crystal structures of related compounds. Subsequent full geometry optimization confirms the structural stability of this phase, as shown in Fig. 1(a). The crystal symmetry analysis shows 16 symmetry operations, including inversion, eight of which include fractional translations. These contain 90° and 180° rotations about crystallographic axes, inversion, and combined inversion–rotation operations. As a result of these symmetry operations, Ce, X, and Y atoms in the tetragonal lattice lead to symmetry-related atomic locations in the unit cell. This structure is characterized by a layered arrangement in which rare-earth and transition-metal pnictide blocks are stacked alternately along the crystallographic *c*-axis. The crystal symmetry is centrosymmetric and belongs to the body-centred tetragonal Bravais lattice. Within this framework, the transition-metal (X) atoms form a square planar network in the ab-plane as shown in Fig. 1(b). The surrounding pnictogen (Y) atoms tetrahedrally coordinate with each X atom, resulting in edge-sharing XY_4_ tetrahedra that extend two-dimensionally throughout the layer (Fig. 1(c)). These tetrahedral units connect to form a robust X–Y network that dominates the in-plane bonding characteristics of the material. The rare-earth Ce atoms occupy the body-centred positions between consecutive X–Y layers (Fig. 1(d)). Fig. 1(e) shows the high-symmetry k-path used for the electronic structure calculations.

Among the CeX_2_Y_2_ compounds, the CeCo_2_Y_2_ exhibit an AFM ordering between the interlayer Co atoms. The Co atoms occupying the same layer couple ferromagnetically with an orientation parallel to the *c*-axis. This in-plane ferromagnetism arises from the larger bond length between the Co atoms (Co–Co) compared to the Co–Y bond length. This stabilizes the intralayer ordering, and the Co–P–Co superexchange results in the inter-layer A-type AFM along the *c*-axis in these compounds.^45–47^ Structurally, the Ce layers act as spacer layers separating the transition-metal–pnictide slabs. This layered arrangement results in strong in-plane covalent bonding within the X–Y sheets and comparatively weaker interlayer interactions along the *c*-direction, imparting a quasi-two-dimensional character to the electronic structure. From an electronic perspective, significant hybridization occurs between the X-3d and Y-3p orbitals, which largely determines the dispersion of the conduction states. In contrast, the Ce-4f states are comparatively localized and contribute prominently to the magnetic properties of the system. The interplay between localized 4f electrons and itinerant 3d electrons plays an important role in defining the magnetic ground state. The layered tetragonal symmetry is crucial for stabilizing antiferromagnetic ordering, as it supports anisotropic magnetic exchange pathways between the Ce moments. Furthermore, the preserved inversion symmetry and specific point-group symmetries of the *I*4/*mmm* structure provide the necessary symmetry conditions to protect characteristic electronic features, including possible band degeneracies and symmetry-enforced crossings in momentum space. To validate the reliability of the present calculations, the obtained lattice parameters are compared with available experimental data. The obtained values of lattice parameters are tabulated in the supplementary section (Table S1) along with the experimentally observed values reported by others. As in the case of CeCo_2_P_2_,^33,34^ the calculated lattice constants show good agreement with experimental values. Similarly, for CeCo_2_As_2_,^34,35^ CeFe_2_P_2_,^33^ and CeNi_2_P_2_,^36^ confirming the accuracy of the computational approach.

### 3.2 Electronic properties

**Fig. 2:**
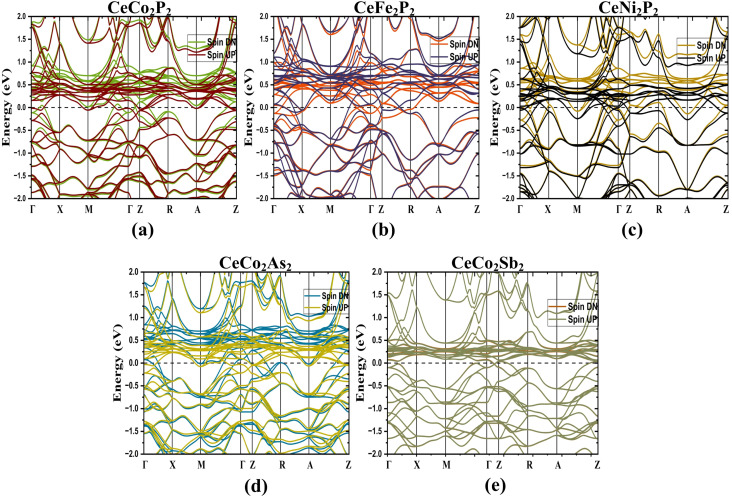
Electronic band structure of tetragonal ThCr_2_Si_2_-type compounds using GGA approach at equilibrium lattice parameters. (a) CeCo_2_P_2_, (b) CeFe_2_P_2_, (c) CeNi_2_P_2_, (d) CeCo_2_As_2_ and (e) CeCo_2_Sb_2_. Spin up (UP) and spin down (DN) states are shown with different colors in each panel.

To study the electronic properties, the BS for the optimized structure is obtained along the high-symmetry paths in the Brillouin zone as shown in Fig. 1(e). Fig. 2(a–e) represents the band structure for spin up and spin down states of CeX_2_P_2_ (X = Fe, Co, and Ni), and CeCo_2_Y_2_ (Y = P, As and Sb), respectively, in the magnetic configuration (ferromagnetic and antiferromagnetic). As in the case of CeX_2_P_2_, it can be seen that CeCo_2_P_2_ has a metallic feature with the Co atoms occupying adjacent layers couple antiferromagnetically, whereas for CeFe_2_P_2_ and CeNi_2_P_2_ such coupling cannot be observed, and the symmetric spin states result in zero moment value at Fe/Ni sites. Also, in all the three compounds, flat bands can be seen above the E_*F*_, which shows the highly localized Ce – f states, and the magnetism in the CeX_2_P_2_ (X = Fe, and Ni) compounds mainly arises from the Ce atom. The energy states above the E_*F*_ are mainly from the Ce atom, and below the *E*_F_, we can see the spin-up and spin-down bands overlapping each other. These overlapping shows the degenerate spin-up and spin-down bands of Co atoms in CeCo_2_P_2_.

For CeCo_2_Y_2_ as shown in Fig. 2(a, d and e), the Co states are coupling antiferromagnetically in all the 3 compounds. Moving from CeCo_2_P_2_ to CeCo_2_Sb_2_, the localized f states are shifting towards the *E*_F_ and this can be due to the change in the lattice parameters due to the change in the size of pnictogen atoms. The Fig. 2(e) clearly indicates that the Ce states have no contribution towards the total magnetic moment and interlayer Co moments are completely compensated, resulting in a completely compensated magnetic configuration or a perfect antiferromagnetic behaviour in CeCo_2_Sb_2_.

**Fig. 3:**
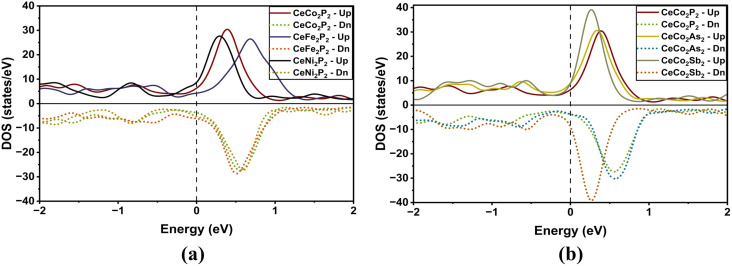
Density of states (DOS) of (a) CeX_2_P_2_ (X = Fe, Co, and Ni), (b). CeCo_2_Y_2_ (Y = P, As and Sb) as a function of energy (in eV). The variation in DOS highlights the influence of transition-metal substitution (Fe, Co, Ni) and pnictogen replacement (P, As, Sb) on the electronic structure near the Fermi level. The vertical dotted line at 0 eV represents the Fermi energy, providing a reference to distinguish occupied and unoccupied states and to assess the metallic nature of the compounds.

To validate the BS plots, we have plotted the DOS for each of the compounds Ce_2_X_2_P_2_ as depicted in Fig. 3(a) and (b). The localized nature of Ce atoms is clear from these plots. The sharp peak near the *E*_F_ in the conduction band (CB) clearly indicates the localized nature of Ce-f electrons. The Co states are mainly found in the valence band (VB) with symmetric spin-up and spin-down electrons. This indicates the antiferromagnetic interaction between the Co atoms. Since Ce-4f electrons are strongly correlated, we further examined using the GGA + *U* approach to account for strong electronic correlations, and the results are tabulated in Table 1. Compared with the conventional GGA results, the inclusion of the Hubbard U parameter mainly shifts the Ce-4f states away from the Fermi level, reducing the hybridization between Ce-4f and Co-d states. However, the overall band topology and the key electronic features near the Fermi level remain largely preserved (see Fig. S1 in the SI). This indicates that the essential electronic properties predicted within GGA are robust against correlation effects. Also, we can see that the calculated lattice parameters and other properties within the GGA framework are in agreement with the experimentally observed results, which suggests the accuracy of the results.

**Table 1:** Calculated values of lattice constants, formation energy (*E*_FE_) and total magnetic moment of CeCo_2_Y_2_ compounds both in GGA as well as GGA + *U*

Compound		*a* (Å)	*c* (Å)	*E*_FE_ (eV)	*M*_T_ (*µ*_B_)
CeCo_2_P_2_	GGA	3.89	9.51	−5.12	0.20
	GGA + *U*	3.95	9.75	−2.58	0.41
CeCo_2_As_2_	GGA	4.04	10.16	−4.51	0.74
	GGA + *U*	4.12	10.04	−4.08	−0.37
CeCo_2_Sb_2_	GGA	4.33	10.73	−2.40	0.00
	GGA + *U*	4.41	10.76	−2.31	−1.73

### 3.3 Magnetic properties

**Fig. 4:**
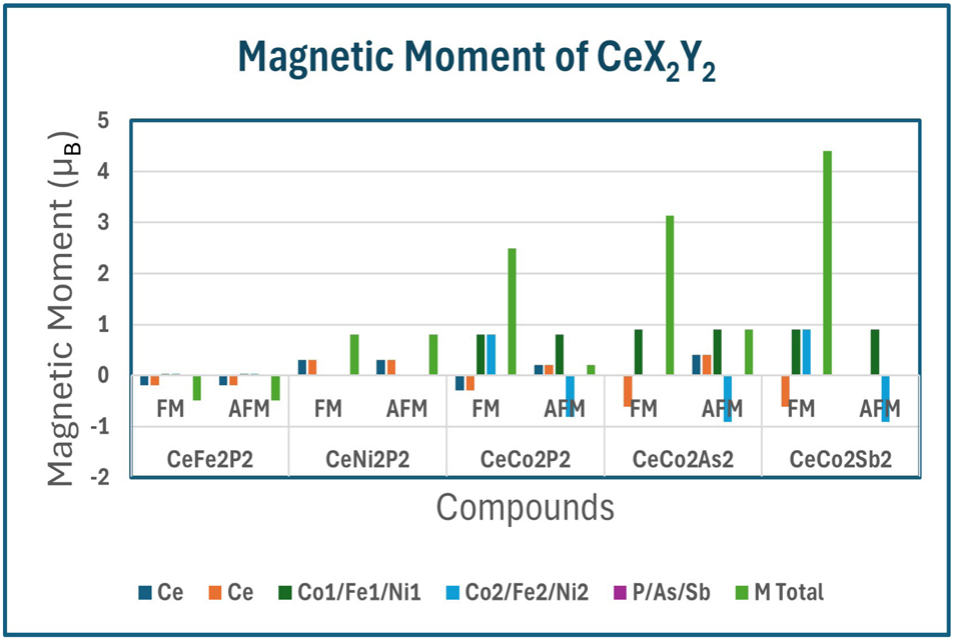
The graphical representation illustrating the variation of individual and total magnetic moment in *µ*_B_ for CeX_2_Y_2_ (X = Fe, Co, and Ni: Y = P, As and Sb) both in ferromagnetic and antiferromagnetic configuration.

To explain the magnetic properties in detail, we have included the individual and total magnetic moments, and the variation of the same is shown in Fig. 4. As seen in the BS and DOS plot, we can see that the total magnetic moment is mainly due to the Ce atoms in CeFe_2_P_2_ and CeNi_2_P_2_, whereas for CeCo_2_Y_2_ the Co moments get compensated and the total magnetic moments come from the uncompensated Ce atoms, which is evident from the asymmetric DOS as given in Fig. 3(a) and (b). It can be seen from Fig. 4 that the magnetic moments increase at Co site as the Co–Co bond increases from CeCo_2_P_2_ to CeCo_2_Sb_2_, which has already been observed for compounds having ThCr_2_Si_2_ type structure like germanides and manganese silicides.^21,48^

The magnetic moments indicate a crossover from weak itinerant magnetism in Fe/Ni systems to robust localized 3d magnetism in Co-based compounds. Increasing pnictogen size enhances moment localization and stabilizes layered antiferromagnetic ordering. Ce moments are induced *via* 3d–4f hybridization and follow transition-metal magnetic ordering, highlighting strong exchange coupling between Ce and Co layers. The Co-based CeCo_2_Y_2_ (Y = P, As, Sb) compounds exhibit robust intralayer ferromagnetic Co ordering combined with interlayer antiferromagnetic stacking. Positive exchange splitting at the Co2 sites leads to a finite positive magnetic moment due to spin-up states in the higher energy region (see Fig. S2 in the SI section), whereas negative exchange splitting at the Co1 sites gives rise to a negative magnetic moment value due to the spin-down states in the higher energy region (see Fig. S2 in the SI section). Increasing pnictogen size reduces 3d–p hybridization, enhances Co moment localization, and strengthens Ce–Co exchange coupling. The induced Ce-4f polarization follows the Co magnetic configuration, evidencing strong 3d–4f interaction. Also, we can see an increase in the magnetic moment moving from CeCo_2_P_2_ to CeCo_2_Sb_2_.

### 3.4 Spin splitting behaviour in CeCo_2_Y_2_ – a possible signature of altermagnetic behaviour

**Fig. 5:**
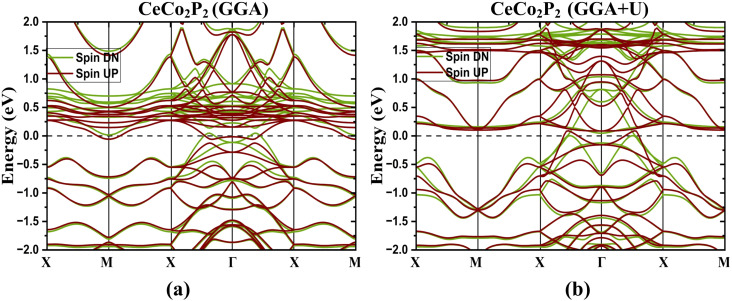
Electronic band structure for CeCo_2_P_2_ along the high symmetry point with (a) GGA and (b) GGA + *U*. In the GGA case, there is a band splitting near the Fermi level, indicating the signature of altermagnetic behaviour. While the incorporation of on-site interaction only modifies the band structure. Here, spin up and spin down states are shown in different colours.

**Fig. 6:**
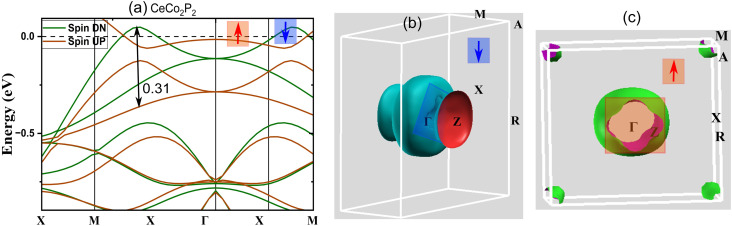
(a) The zoomed version of the electronic band structure of the CeCo_2_P_2_ within GGA near the Fermi level showing altermagnetic spin splitting. Here, the maximum splitting is of the order of 0.31 eV along the X–Γ–X direction. (b) The Fermi surface plot for spin down (↓) states, and (c). for the spin up (↑) states depicting the d-wave altermagnetic behaviour in CeCo_2_P_2_.

**Fig. 7:**
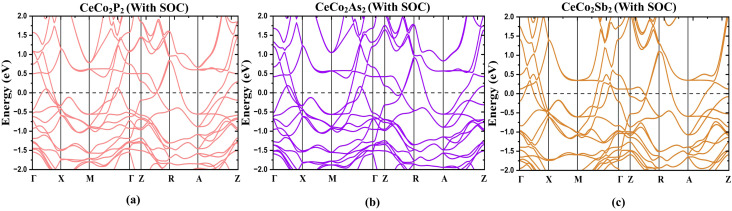
Electronic band structures of CeCo_2_Y_2_ calculated with the inclusion of spin–orbit coupling (SOC). Panels correspond to Y = P, As, and Sb, illustrating the influence of SOC on the band dispersion near the Fermi level. The Fermi energy is set at 0 eV.

As proposed by Sakhya *et al.*, the CoNb_4_Se_8_ displays an antiferromagnetic ordering of the A-type, in which the Co planes are stacked antiferromagnetically along the *c*-axis yet ferromagnetically coupled in-plane, meets the condition for altermagnetism.^23^ Therefore, from the previous discussion, it is evident that the proposed CeCo_2_Y_2_ compounds show A-type AFM and thus these layered antiferromagnets can be probed for the existence of altermagnetic band splitting. Also, the presence of heavier elements paves a way to study the effect of the spin–orbit coupling in these compounds. The band structure plot, as depicted in Fig. 2(a, d and e), shows an indication of band splitting along the high symmetry points. To delve deeper into this for more evidence, the band structure plot is shown in a different k-path in Fig. 5 where the splitting is more evident. Here we have plotted the band structure in both GGA (Fig. 5(a)) and GGA + U (Fig. 5(b)) approximations. From the figure, it is clear that the splitting in CeCo_2_P_2_ is much more evident along the X–Γ–X direction. To obtain the magnitude of splitting, the zoomed figure is shown in Fig. 6(a). We have obtained a maximum band splitting of 0.31 eV for this compound. To validate the claim, we have plotted the Fermi surface for CeCo_2_P_2_ for both spin up and spin down and depicted in Fig. 6(b) and (c). From the figure, we can observe a four-lobed clover-like structure, and this suggests that the CeCo_2_P_2_ has a d-wave altermagnetic splitting. The splitting is prominent along the X–Γ–X path as mentioned before, and as we look into the Fermi surface for spin-up and spin-down (Fig. 6(b) and (c)), we can see that the d-wave splitting is along the same k-path direction. For spin-down states, we can see an extra circular surface, which is above the X–Γ–X plane and can be a contribution from a different k-path direction. For CeCo_2_As_2_ and CeCo_2_Sb_2_, we can see that the degeneracy of spin-up and spin-down bands are getting slightly removed for the former, and for the latter, the material is completely antiferromagnetic. This change may be due to the influence of localized flat bands of Ce atoms, as it is evident from the Fig. 3. As a result, CeCo_2_P_2_ can be chosen for exploring the alter-magnetism in Co-based pnictides. For CeCo_2_Y_2_, the electronic BS, including SOC are calculated along the high-symmetry directions of the tetragonal Brillouin zone and is presented in Fig. 7. The fact that several bands cross the E_*F*_ suggests that these compounds are metallic. Because of the relativistic effects related to Ce-4f electrons, the presence of SOC causes a slight splitting of degenerate bands, especially close to the Γ and M points. Additionally, as the atomic number of the pnictogen element increases, the SOC-induced splitting becomes more noticeable from CeCo_2_P_2_ to CeCo_2_Sb_2_. Near 0.1 eV, two bands in CeCo_2_P_2_ and CeCo_2_As_2_ approach one another with nearly linear dispersion, which may be an indication of Dirac-like crossover. However, SOC probably creates a tiny gap, which means a Dirac feature with a SOC-induced gap. Near the Fermi level, a number of band crossings are seen in the Γ–X and Γ–Z directions. In the absence of SOC, these crossings point to potential Dirac-like characteristics. A small amount of band splitting occurs when SOC is added, suggesting that relativistic effects lift the degeneracy. For CeCo_2_Sb_2_ near M, we can see almost flat as well as dispersive band interaction. This often arises from Ce-4f hybridization with Co-3d orbitals and cannot usually form a topological Dirac point.

## 4 Conclusions

The DFT studies were carried out on CeX_2_Y_2_ compounds within the GGA approach by adopting the experimentally reported ThCr_2_Si_2_-type structure phase, which is widely known for this class of materials (space group – *I*4/mmm). Electronic results show a metallic nature of the compounds, showing highly localized f electrons of Ce atoms near *E*_F_. The localized nature of Ce-f electrons is further confirmed through the density of states, and Co states are mainly found in the valence band. The correlation effects studied after including GGA + U show a shift in the electronic states. However, the overall band topology and the key electronic features near the Fermi level remain largely preserved. This indicates that the essential electronic properties predicted within GGA are robust against the correlation effect. The magnetic studies show weak magnetism for X = Fe and Ni compounds, whereas for Co compounds, the interlayer Co atoms are coupled antiferromagnetically and make these compounds interesting. The projected density of states indicates that positive exchange splitting at Co2 sites leads to positive magnetic moments, whereas negative exchange splitting at Co1 sites results in negative magnetic moment values. The spin projected band with GGA revealed that CeCo_2_P_2_ shows momentum-dependent spin splitting of 0.31 eV along the X–Γ–X high symmetry direction and hence, CeCo_2_P_2_ exhibit altermagnetic behaviour. The spin-resolved Fermi surface plots show a four lobe structure which indicates a d-wave-like splitting of the bands. Finally, on taking into account the spin–orbit coupling, the small amount of band splitting occurs, suggesting that relativistic effects lift the degeneracy. These results establish the studied rare-earth-based compound as an effective platform for understanding the interplay of structural, magnetic, and spin–orbit coupling–driven phenomena. Furthermore, CeCo_2_P_2_ is identified as a d-wave altermagnet, highlighting its potential for applications in next-generation spintronic devices and symmetry-driven electronic technologies.

## Author contributions

Narayanan Namboodiri Puthusseri: conceptualization, methodology, investigation, visualization, formal analysis, data curation, writing – original draft. Sambit Jena: formal analysis, data curation. Tanay Nag: writing – review and editing, formal analysis, conceptualization. Banasree Sadhukhan: writing – review and editing, formal analysis, project administration, funding acquisition, conceptualization. Pankaj Bhalla: supervision, writing – review and editing, project administration, funding acquisition, formal analysis, conceptualization.

## Conflicts of interest

The authors declare that there are no conflicts of interest related to this work.

## Supplementary Material

RA-016-D6RA02985J-s001

## Data Availability

The data that support the findings of this study are available from the corresponding author upon reasonable request. Supplementary information (SI) is available. See DOI: https://doi.org/10.1039/d6ra02985j.
