# Supplementary material for: Transition metal driven altermagnetism and spin–orbit coupling effect in tetragonal Ce-based pnictides: a first-principles investigation
Source: RSC Adv. 2026 May 26;16(31):28405–12. doi: 10.1039/d6ra02985j (PMC13213574; doi:10.1039/d6ra02985j)
Supplement: RA-016-D6RA02985J-s001 [file RA-016-D6RA02985J-s001.pdf]

Supplementary Information (SI) for RSC Advances.
This journal is © The Royal Society of Chemistry 2026

Supporting Information for

Transition Metal Driven Altermagnetism and Spin–Orbit Coupling Effect in Tetragonal Ce-Based Pnictides: A First-Principles Investigation

Narayanan Namboodiri Puthusseri[a], Sambit Jena[b], Tanay Nag[c], Banasree Sadhukhan[b] and Pankaj Bhalla[a*]

[a]Department of Physics, School of Engineering and Sciences, SRM University AP, Amaravati 522240, India
[b]Department of Physics, École Centrale School of Engineering, Mahindra University, Hyderabad, Telangana 500043, India.
[c]Department of Physics, BITS Pilani-Hyderabad Campus, Telangana 500078, India.

*Corresponding author: Pankaj Bhalla: pankaj.b@srmap.edu.in

1 Formation Energy

The formation energy (E_{FE}) has been calculated using the given formula for each compound, and the calculated values are tabulated in Table S1.

$$E_{FE} = E_{\mathrm{CeX_2Y_2}} - [E_{Ce} + 2E_X + 2E_Y] \tag{1}$$

where E_{FE} is the calculated formation energy, $E_{\mathrm{CeX_2Y_2}}$ represents the energy of bulk $CeCo_2Y_2$ and E_{Ce}, E_{Co}, E_Y, respectively represents the energy of individual Ce, X and Y atoms.

Table S1: Calculated values of lattice constants obtained for the present work, experimentally reported lattice constants, total energy and formation energy (E_{FE}) of CeX_2Y_2 compounds with GGA-PBE approximation.

Compound	This Work		Experimental		Energy (eV)	Formation Energy (eV)
	a (Å)	c (Å)	a (Å)	c (Å)		
$CeFe_2P_2$	3.86	9.53	3.852 [1]	10.31 [1]	-31810.45	-5.25
$CeNi_2P_2$	3.92	9.55	3.955 [2]	9.505 [2]	-32573.54	-7.69
$CeCo_2P_2$	3.89	9.51	3.895 [1]	9.604 [1]	-30135.92	-5.12
$CeCo_2As_2$	4.04	10.16	4.026 [3]	10.216 [3]	-30358.87	-4.51
$CeCo_2Sb_2$	4.33	10.73			-39430.75	-2.40

The tabulated values of lattice constants show that the lattice constants obtained after full geometry optimization are in agreement with the experimentally reported works. The negative value of formation energy ascertain the structural stability of the investigated compounds.

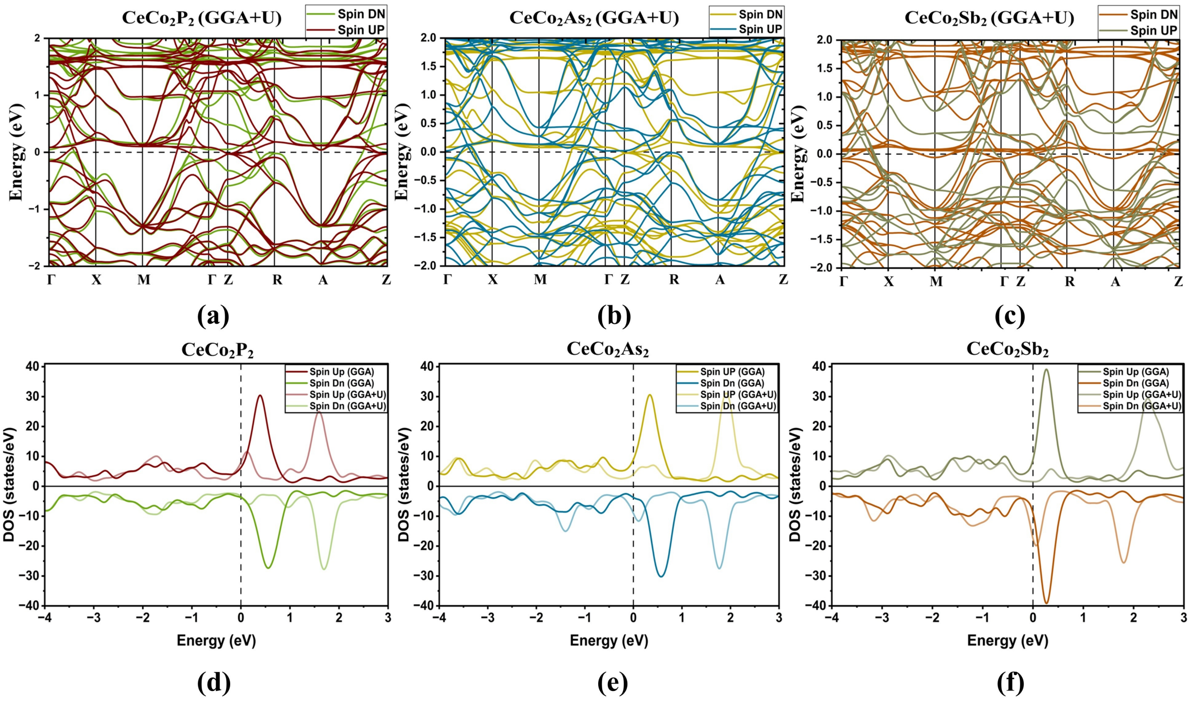

Fig. S1: (a)–(c). Electronic band structures and (d)–(f). corresponding density of states (DOS) of $CeCo_2Y_2$ (Y = P, As, and Sb) obtained using the DFT+U method. The on-site Coulomb interaction (U) is included for the Ce-4f and Co-3d states to account for correlation effects. Panels (a,d), (b,e), and (c,f) represent Y = P, As, and Sb, respectively. The Fermi level is set at 0 eV.

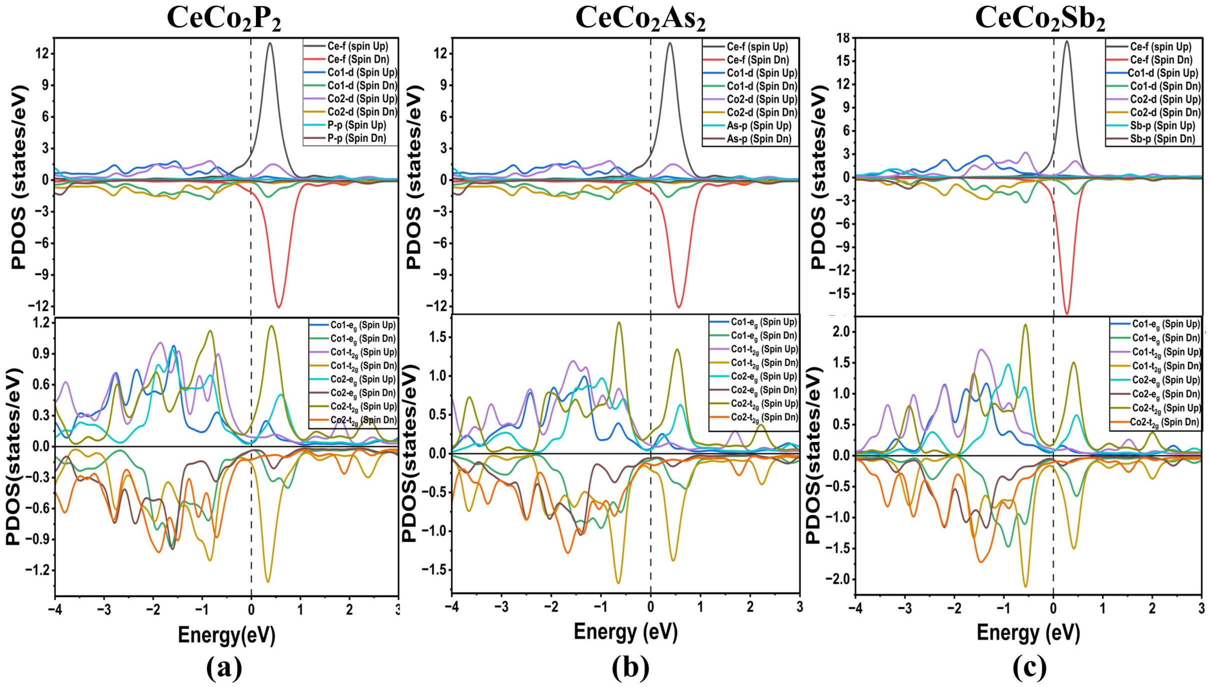

Fig. S2: The projected density of states (PDOS) for Ce-based compounds computed within GGA (a). $CeCo_2P_2$, (b). $CeCo_2As_2$, (c). $CeCo_2Sb_2$. The top panel of the plot represents the orbital projections of Ce-f, Co-d and P-p orbitals, and the bottom panel represents PDOS of e_g and t_{2g} levels of Co1 and Co2 d orbitals for $CeCo_2X_2$ compounds.

References

1 M. Reehuis and W. Jeitschko, *J. Phys. Chem. Solids*, 1990, **51**, 961–968.

2 W. K. Hofmann and W. Jeitschko, *J. Solid State Chem.*, 1984, **51**, 152–158.

3 C. M. Thompson, X. Tan, K. Kovnir, V. O. Garlea, A. A. Gippius, A. A. Yaroslavtsev, A. P. Menushenkov, R. V. Chernikov, N. Büttgen, W. Krätschmer, Y. V. Zubavichus and M. Shatruk, *Chem. Mater.*, 2014, **26**, 3825–3837.
